# Changes in plasma CXCL4 levels are associated with improvements in lung function in patients receiving immunosuppressive therapy for systemic sclerosis-related interstitial lung disease

**DOI:** 10.1186/s13075-016-1203-y

**Published:** 2016-12-30

**Authors:** Elizabeth R. Volkmann, Donald P. Tashkin, Michael D. Roth, Philip J. Clements, Dinesh Khanna, Daniel E. Furst, Maureen Mayes, Julio Charles, Chi-Hong Tseng, Robert M. Elashoff, Shervin Assassi

**Affiliations:** 1 Department of Medicine, University of California, David Geffen School of Medicine, Los Angeles, CA USA; 2 Department of Medicine, University of Michigan Medical School, Ann Arbor, MI USA; 3 Department of Medicine, University of Texas Health Science Center at Houston, Houston, TX USA; 4 Department of Biomathematics, University of California, Los Angeles, CA USA

**Keywords:** Systemic sclerosis, Pulmonary fibrosis, Chemokines, Immunosuppression

## Abstract

**Background:**

Increased circulatory levels of the chemokine CXCL4 have been associated with the presence of interstitial lung disease (ILD) in an observational study of patients with systemic sclerosis (SSc). The purpose of the present study was to evaluate the relationship between baseline CXCL4 level and extent of ILD in the context of a randomized controlled trial and to determine whether changes in CXCL4 levels in response to immunosuppression are associated with future progression of SSc-ILD.

**Methods:**

A total of 142 SSc-ILD patients from Scleroderma Lung Study (SLS) II were randomized in a double-blind, parallel-arm trial, to receive mycophenolate (MMF) for 2 years or oral cyclophosphamide (CYC) for 1 year followed by 1 year of placebo. Plasma CXCL4 levels were measured at baseline, 12 months, and 24 months in SLS II participants (*N* = 136) and at a single time point in healthy controls (*N* = 67). A mixed-effects model evaluated the relationship between change in CXCL4 levels and SSc-ILD progression. The primary outcome was the course of the forced vital capacity.

**Results:**

Baseline CXCL4 levels were significantly higher in SSc-ILD patients compared with healthy controls (2699 ± 1489 ng/ml vs 2233 ± 1351 ng/ml (mean ± SD); *P* = 0.019). However, no significant correlations were identified between CXCL4 levels and extent of ILD at baseline, as measured by the forced vital capacity, diffusing capacity of carbon monoxide, or radiographic extent of ILD. Plasma CXCL4 decreased significantly from baseline to 12 months in all patients (CYC: *P* < 0.001; MMF: *P* = 0.006) with no between-treatment differences (CYC vs MMF). Patients with the largest decline in CXCL4 levels during the first 12 months had an improved course of forced vital capacity %-predicted from 12 to 24 months (*P* = 0.040), even after adjusting for baseline disease severity and treatment arm assignment.

**Conclusions:**

Levels of CXCL4 were higher in patients with SSc-ILD compared with controls and decreased in all patients treated with immunosuppressive therapy. While CXCL4 levels were not correlated with extent of ILD at baseline, changes in CXCL4 at 12 months predicted future progression of SSc-ILD from 12 to 24 months. These findings suggest that intermediate-term changes in CXCL4 may have predictive significance for long-term progression of SSc-ILD in patients receiving immunosuppressive therapy.

**Trial registration:**

ClinicalTrials.gov NCT00883129. Registered 16 April 2009.

## Background

Although interstitial lung disease (ILD) occurs in the majority of patients with systemic sclerosis (SSc) and is also the leading cause of death in SSc [1], ILD progression rates vary considerably in this patient population [2].

Identifying specific factors that may predict response to immunosuppression is central to improving our ability to determine which SSc patients may derive the greatest benefit from ILD-targeted therapy. While numerous studies have examined baseline factors that are associated with ILD progression [3–9], relatively few studies have examined whether these factors are associated with a positive response to immunosuppression. In the Scleroderma Lung Study (SLS) I (comparing cyclophosphamide (CYC) with placebo), the severity of reticular infiltrates on baseline high-resolution computed tomography (HRCT) was associated with improved responsiveness to CYC therapy [10]. However, to our knowledge, no studies have examined responsiveness to therapy with mycophenolate (MMF) in SSc-ILD.

Biomarkers may have clinical application for predicting response to immunosuppression in patients with SSc-ILD. Several studies have identified potential circulating biomarkers associated with accelerated SSc-ILD progression, including interleukin (IL)-6 [11], C-reactive protein (CRP) [12], monocyte chemoattractant protein-1 (MCP-1) [13], CC chemokine ligand 18 (CCL18) [14, 15], and CXCL4 [16]. However, these markers have not been consistently shown to predict relevant clinical outcomes across a variety of treatments and SSc populations. Furthermore, all of the studies assessing the aforementioned candidate biomarkers were observational by design and consequently included patients who exhibited diverse phenotypic expressions of SSc, received a variety of immunosuppressant therapies, and were followed for varying periods of time [11–16].

Of these potential biomarkers, the chemokine, CXCL4, may play an important role in perpetuating profibrotic activity in the SSc-ILD disease state. An observational study found that CXCL4 levels were higher in SSc patients with ILD compared with those without ILD in a discovery cohort, as well as in two independent replication cohorts [16]. In a subgroup of patients from this study (*N* = 79), SSc patients with high baseline CXCL4 levels had accelerated progression of ILD as measured by a decline in the diffusing capacity for carbon monoxide (DLCO) [16]. Notably, this study also found that CXCL4 levels were remarkably higher in SSc patients compared with healthy controls (270-fold higher) [16].

The present study aimed to evaluate whether the chemokine CXCL4 is associated with the extent of ILD in a well-characterized cohort of SSc patients, all of whom had clinically significant ILD. Unlike previous studies of this nature, the present study analyzed CXCL4 at multiple time points in concert with the simultaneous collection of extensive clinical, physiologic, and radiographic data. A secondary aim was to determine whether changes in CXCL4 levels can predict improvements in lung function in patients receiving MMF or CYC in the setting of a randomized controlled clinical trial (RCT).

## Methods

### Study participants

Participants in the SLS II trial (*N* = 142) were all adults, aged 18–75 years, who exhibited either limited or diffuse cutaneous SSc [17] and active ILD as demonstrated by restrictive ventilatory impairment (forced vital capacity (FVC) <80% but ≥45% predicted), exertional dyspnea (Grade ≥2 on the Magnitude of Task component of the Mahler Baseline Dyspnea Index (BDI)) [18], and the presence of any ground glass opacity (GGO; hazy opacity through which normal lung markings can be discerned) on HRCT. Key exclusion criteria included pulmonary hypertension; clinically significant abnormalities on HRCT not attributable to SSc; and smoking within the past 6 months or evidence of significant airflow obstruction. Complete details of the SLS II population have been reported previously [19].

Healthy control participants were independently recruited at the University of Texas, Houston, and matched for age, ethnicity, and gender to SLS II participants in a 1:2 ratio.

### SLS II study design

Enrolled patients were randomly (1:1 block) assigned in a double-blind, parallel-arm manner to receive either oral CYC for 1 year followed by 1 year of placebo or MMF for 2 years. SLS II was a superiority study conducted at 14 university hospitals throughout the USA (for the list of sites see Ethics approval and consent to participate) and recruited patients between November 2009 and January 2013. For complete details of the SLS II protocol, please see the supplementary Web appendix accompanying the main SLS manuscript [19]. There were no significant changes in the protocol involving the design, outcomes, treatment, or eligibility criteria during the trial. Baseline measurements included the following physiological variables: spirometry (FVC and forced expired volume in 1 second (FEV_1_)), lung volumes (functional residual capacity (FRC), residual volume (RV), and total lung capacity (TLC) by whole-body plethysmography or helium dilution), hemoglobin-adjusted single-breath DLCO, and the ratio of DLCO to alveolar volume (DL/VA). The FVC (primary SLS II endpoint) and DLCO (secondary SLS II endpoint) were measured every 3 months, and the TLC was measured every 6 months during the trial. HRCT thoracic imaging was obtained at baseline and at 24 months and a computer-aided design (CAD) scoring system was employed to provide quantitative measures of different patterns of ILD as described previously [19, 20]. The Quantitative ILD (QILD) score was the sum of all abnormally classified scores, including scores for quantitative lung fibrosis (QLF, linear reticular markings), GGO, and honeycomb changes (clustered air-filled cysts with dense walls). Scores were calculated as the percentage of voxels for both the whole lung (WL), including both lungs, and for the lobe of maximal involvement (LM).

### CXCL4 measurement

SLS II plasma samples were collected at the baseline, 12-month, and 24-month study visits and were immediately processed on-site on the day of collection, stored at −70 °C, and shipped on dry ice to the central repository at the University of Texas, Houston. Samples from healthy controls collected at the University of Texas, Houston, were handled in the same manner except that no shipping was required. Validated, commercially available ELISA kits (Human CXCL4/PF4 Quantikine Kit; R&D Systems) were used to assess CXCL4 levels. All assays were performed in duplicate and the coefficient of variance was <20%. Technicians performing the assays were blinded to the study arm assignment. CXCL4 data were logarithmically transformed to correct for data skewness.

### Statistical analysis

#### Baseline characteristics

Summary statistics were generated for baseline characteristics. A two-sample *t* test was used to compare continuous variables and a chi-square test was used to compare categorical variables. Pearson correlations were performed to examine the relationship between CXCL4 levels and baseline measures of extent of ILD, as measured by the FVC, DLCO, QILD, and QLF, as well as the relationship between baseline CXCL4 levels and platelet counts.

#### Relationship between change in CXCL4 and progression of SSc-ILD

To determine whether a change in plasma CXCL4 might correlate with or predict the progression of SSc-ILD, we first plotted the course of measured plasma CXCL4, by treatment arm, from baseline to 24 months and determined the corresponding magnitude of change for individual subjects from baseline to 12 and 24 months. Pearson correlations were initially performed to examine the relationship between the change in CXCL4 levels from baseline to 12 months and the absolute change for the primary outcome (FVC %-predicted) and key secondary outcomes (DLCO %-predicted and HRCT measures of the extent of ILD).

Following these initial analyses, a mixed-effects model was generated, the outcome of which was the course of FVC %-predicted measured in 3-month increments from 12 to 24 months (primary outcome). The model included the following covariates: change in CXCL4 from baseline to 12 months, baseline FVC %-predicted, baseline extent of quantitative fibrosis/ILD, treatment arm, and a time trend. The covariate of baseline extent of quantitative fibrosis/ILD was defined as the first principal component from a principal component analysis of the following variables: QILD-LM, QILD-WL, QLF-LM, and QLF-WL.

All tests were two-sided, and all analyses were performed using SAS 9.2 (SAS Institute, Inc., Cary, NC, USA).

## Results

### Baseline characteristics

**Fig. 1:**
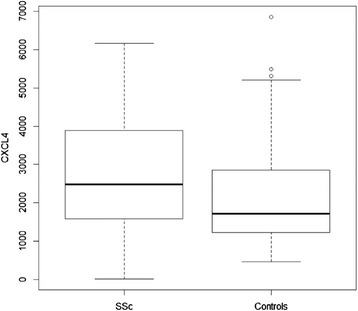
Plasma CXCL4 levels (ng/ml) were significantly higher in SSc-ILD patients (*N* = 136) compared with healthy controls matched for age, gender, and ethnicity (*N* = 67), *P* = 0.019. *SSc* systemic sclerosis

At baseline, there were no significant differences in any of the demographic or key disease-defining characteristics of SLS II participants assigned to MMF vs CYC (Table 1). Baseline CXCL4 levels (ng/ml) were higher in SLS II participants (*N* = 136) compared with healthy controls (*N* = 67) (SSc: mean 2699 (SD 1489), median 2480 (IR 1585, 3889); controls: mean 2233 (SD 1351), median 1720 (IR 1224, 2874); *P* = 0.019) (Fig. 1). There were no significant differences in baseline CXCL4 levels between treatment arms, between limited or diffuse SSc patients, between men and women, or between ethnicities (all *P* ≥ 0.2). There were also no significant correlations between baseline CXCL4 and age, or disease duration, as measured by the onset of the first Raynaud’s phenomenon symptom (*r* = −0.10, *P* = 0.24), or as measured by the onset of the first non-Raynaud’s phenomenon symptom attributable to SSc (*r* = 0.040, *P* = 0.64). Similarly, among the healthy controls there were no significant differences in CXCL4 levels between men and women or between ethnicities, and nor was there any correlation between CXCL4 levels and age.

**Table 1:** Baseline characteristics of SLS II participants with CXCL4 measurements at baseline

	Cyclophosphamide		Mycophenolate	
Characteristic	*N*		*N*	
Age (years)	71	52.3 ± 9.5	65	52.6 ± 10.0
Female sex (% of patients)	71	77.5	65	69.2
Duration of scleroderma (years)	70	2.5 ± 1.8	63	2.7 ± 1.7
% Limited/% diffuse	71	45.1/54.9	65	36.9/63.1
FVC (%-predicted)	71	66.2 ± 9.9	65	66.6 ± 8.2
FEV1:FVC (%-predicted)	71	83.5 ± 5.6	65	81.8 ± 5.7
TLC (%-predicted)	71	65.4 ± 12.1	65	66.5 ± 10.2
DLCO (%-predicted)^a^	71	53.8 ± 14.2	65	54.4 ± 11.3
DL/VA (%-predicted)	71	61.0 ± 13.7	65	60.9 ± 11.8
Mahler Dyspnea Index (focal score)	67	7.0 ± 2.3	61	7.3 ± 2.1
Skin-thickening score (mRSS)^b^				
All patients	71		65	
Mean		14.1 ± 10.8		15.2 ± 10.1
Range		2–46		1–41
Patients with dcSSc	39		41	
Mean		20.7 ± 9.9		20.7 ± 8.6
Range		3–46		4–41
Patients with lcSSc	32		24	
Mean		6.1 ± 4.3		5.9 ± 3.4
Range		2–18		1–14
HAQ disability index (0–3)^c^	71	0.7 ± 0.7	65	0.7 ± 0.6
QLF-WL	69	9.1 ± 7.0	62	8.2 ± 6.9
QLF-LM	69	23.2 ± 19.2	62	22.5 ± 19.9
QILD-WL	69	32.1 ± 14.2	62	27.2 ± 13.6
QILD-LM	69	53.2 ± 19.3	62	49.8 ± 21.0
Auto-antibody (% positive in patients tested)				
ANA	70	94.3	62	96.8
Topoisomerase-1	70	45.7	62	46.2
RNA polymerase III	70	11.4	62	14.5
Centromere	70	2.9	62	1.6
Th\to	67	7.5	60	5.0

Values are mean ± standard deviation, unless otherwise noted

^a^Adjusted for hemoglobin

^b^Scores for skin thickening (mRSS) can range from 0 to 51, with higher scores indicating more severe thickening

^c^Scores for HAQ Disability Index can range from 1 to 3, with higher numbers indicating greater disability

*FVC* forced vital capacity, *FEV*_*1*_ forced expired volume in 1 second, *TLC* total lung capacity, *DLCO* diffusing capacity of the lung for carbon monoxide, *DL/VA* ratio of DLCO to alveolar volume, *mRSS* modified Rodnan skin score, *dcScc* diffuse cutaneous systemic sclerosis, *lcSSc* limited or cutaneous systemic sclerosis, *HAQ* Health Assessment Questionnaire, *QLF* quantitative extent of lung fibrosis on high-resolution chest computed tomography, *QILD* quantitative extent of total interstitial lung disease (including fibrosis, honeycomb, and ground glass opacity), *WL* whole lung, *LM* lobe of maximal involvement

*ANA* Anti-nuclear antibody, *Th/To* Th/To ribonucleoprotein antibody

### CXCL4 correlations

At baseline there were no significant correlations between plasma CXCL4 levels and any of the physiologic or radiographic measures of extent or severity of SSc-ILD (Table 2). There were also no significant correlations between plasma CXCL4 and extent of SSc-ILD at 12 or 24 months (data not shown).

**Table 2:** Baseline CXCL4 levels are not correlated with extent of ILD in SLS II subjects

Surrogate ILD measurements	Pearson correlation (*r*)	*P* value
TLC% predicted	0.123	0.154
FVC% predicted	0.089	0.304
DLCO% predicted	0.082	0.345
QLF-LM	−0.027	0.763
QILD-LM	−0.063	0.473
QLF-WL	−0.034	0.703
QILD-WL	−0.022	0.799

Values are mean ± standard deviation, unless otherwise noted

*ILD* interstitial lung disease, *SLS* Scleroderma Lung Study, *FVC* forced vital capacity, *TLC* total lung capacity, *DLCO* diffusing capacity of the lung for carbon monoxide, *QLF* quantitative extent of lung fibrosis on high-resolution chest computed tomography, *QILD* quantitative extent of total interstitial lung disease (including fibrosis, honeycomb, and ground glass opacity), *WL* whole lung, *LM* lobe of maximal involvement

There was no significant correlation between baseline CXCL4 and baseline mRSS among all participants (*r* = 0.10; *P* = 0.24), or among patients with diffuse cutaneous sclerosis (*N* = 80; *r* = 0.059; *P* = 0.60).

At baseline, CXCL4 levels demonstrated a weak but significant correlation with the platelet count (*r* = 0.320, *P* < 0.001).

### Plasma levels of CXCL4 levels decrease in response to immunosuppression

**Fig. 2:**
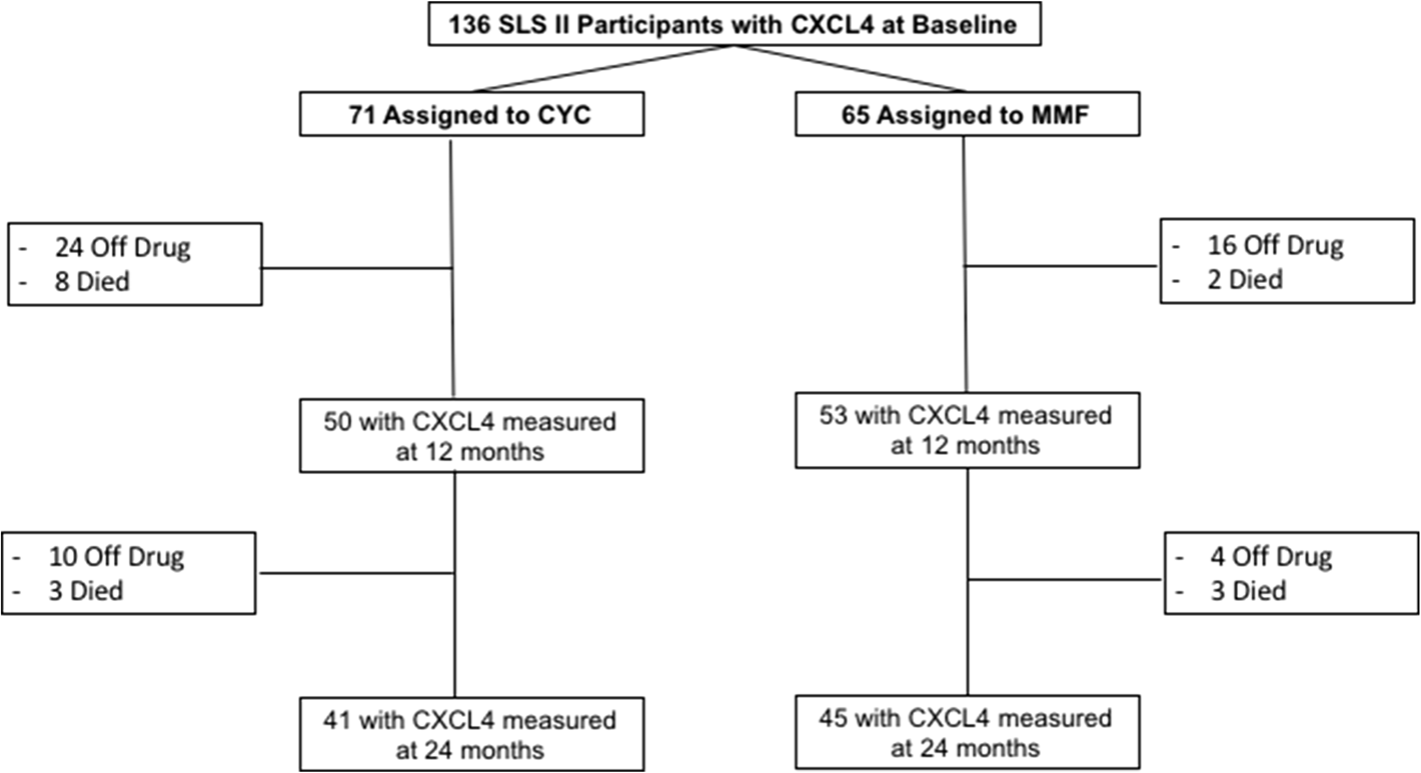
Disposition of SLS II study participants with regard to CXCL4 measurement during the 24-month trial. FVC measurements were obtained in 51 and 59 participants at 12 months and in 50 and 53 participants at 24 months for the CYC and MMF arms, respectively. *CYC* cyclophosphamide, *MMF* mycophenolate, *SLS* Scleroderma Lung Study

Among the 136 patients with baseline plasma CXCL4 levels measured, 103 patients had 12-month CXCL4 measurements (CYC = 50; MMF = 53), and 86 patients had 24-month CXCL4 measurements (CYC = 41; MMF = 45) (Fig. 2). CXCL4 levels were measured in all patients who returned for the follow-up study visits at 12 and 24 months.

**Fig. 3:**
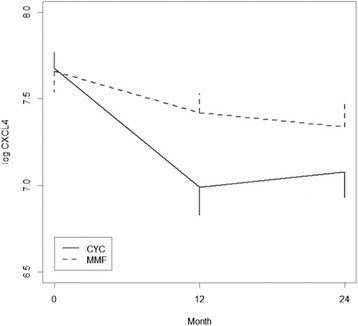
CXCL4 levels decreased significantly in response to immunosuppression in each treatment arm from baseline to 12 months (CYC: *P* < 0.001; MMF: *P* = 0.006) and from baseline to 24 months (CYC: *P* < 0.001; MMF: *P* = 0.011). There was no between-treatment difference in the reduction in CXCL4 from baseline to 12 months or from baseline to 24 months. *CYC* cyclophosphamide, *MMF* mycophenolate

The majority of patients in both treatment arms experienced a decrease in CXCL4 levels from baseline to 12 months (CYC: 76% (37/49); MMF: 67% (35/52)) and from baseline to 24 months (CYC: 70% (28/40); MMF: 68% (30/44)). Plasma CXCL4 levels declined significantly in the CYC and MMF arms (*P* < 0.001 and *P* = 0.006, respectively). Similarly, CXCL4 levels showed a significant decrease from baseline to 24 months in the CYC and MMF arms (*P* < 0.001 and *P* = 0.011, respectively). Although participants in the CYC arm experienced numerically larger decreases in CXCL4 levels, there was no statistically significant difference in the change in CXCL4 levels between treatment arms from baseline to 12 months (*P* = 0.120) or from baseline to 24 months (*P* = 0.380) (Fig. 3). While CXCL4 levels slightly increased from 12 to 24 months in the CYC arm (during the placebo phase), CXCL4 levels continued to decline from 12 to 24 months in the MMF arm.

### Changes in plasma levels of CXCL4 levels may predict improved course of FVC

As reported previously [19], the course of the FVC in SLS II improved significantly from baseline to 24 months in both the CYC and MMF treatment arms, with no between-treatment difference. Of note, FVC measurements were obtained in 51 and 59 participants at 12 months and in 50 and 53 participants at 24 months for the CYC and MMF arms, respectively. When changes in the plasma CXCL4 levels at 12 months were correlated with changes in the FVC (bivariate analysis), there was a trend for a negative correlation between the change in CXCL4 from baseline to 12 months and the change in FVC from baseline to 18 months (*r* = −0.203; *P* = 0.063), 21 months (*r* = −0.212; *P* = 0.062), and 24 months (*r* = −0.182; *P* = 0.082), but not at 12 months (*r* = −0.070; *P* = 0.499) or 15 months (*r* = −0.048; *P* = 0.662).

There were no significant correlations between the change from baseline in CXCL4 at 12 months and the change from baseline in DLCO at 12, 15, 18, 21, or 24 months (all *P* > 0.3). There was also no significant correlation between the change from baseline in CXCL4 at 12 months and the change from baseline in QLF-LM/WL or QILD-LM/WL at 24 months (all *P* > 0.4).

To further explore the relationship between the change in CXCL4 and the course of the FVC, a mixed-effects model was generated and the results demonstrated that the change in CXCL4 from baseline to 12 months was associated with the course of the FVC (as a continuous variable) from 12 to 24 months (estimate −1.232; 95% CI −2.405, −0.060; *P* = 0.040), after controlling for treatment arm assignment, a time trend, baseline quantitative extent of ILD/fibrosis, and baseline FVC. A similar statistical approach was used in the analysis of the primary and secondary outcomes in SLS II [19]. The results demonstrated that every 1-unit decrease in the log CXCL4 level was associated with a 1.23% increase in the FVC. In other words, patients who experienced the greatest decline in CXCL4 levels during the first 12 months of the study had an improved course of FVC from 12 to 24 months (Table 3).

**Table 3:** Multivariable analysis examining the relationship between change in CXCL4 from baseline to 12 months and the course of FVC from 12 to 24 months (*N* = 97)

Baseline covariate	Estimate	95% CI	*P* value
ΔCXCL4	−1.232	−2.405, −0.060	0.040
Baseline FVC	0.972	0.807, 1.138	<0.0001
Baseline QILD/QLF^a^	0.297	−0.496, 1.089	0.464
Treatment arm	−0.361	−3.182, 2.460	0.802

^a^Defined as the first principal component from a principal component analysis of the following variables: QILD-LM, QILD-WL, QLF-LM, and QLF-WL

*FVC* forced vital capacity, *CI* confidence interval, *QLF* quantitative extent of lung fibrosis on high-resolution chest computed tomography, *QILD* quantitative extent of total interstitial lung disease (including fibrosis, honeycomb, and ground glass opacity), *WL* whole lung, *LM* lobe of maximal involvement

Given the significant correlation between CXCL4 levels and platelet count at baseline, baseline platelet count was added to the mixed-effects model. Adding baseline platelet count to the model did not affect the significant relationship between change in CXCL4 levels and the outcome variable. However, the baseline platelet count was not independently associated with the course of the FVC (*P* = 0.163), and for this reason this variable was not included in the final model.

Because not all patients had CXCL4 measurements at 12 months, the final model included 97 patients with SSc-ILD. Notably, there were no significant differences in baseline characteristics (i.e., age, gender, diffuse/limited, FVC, DLCO, MRSS, QILD-WL/LM, QLF-WL/LM) between the 97 patients included in the final mixed-effects model and the 39 patients who were not included.

## Discussion

In a well-characterized cohort of patients with SSc-ILD, CXCL4 levels decreased significantly in response to immunosuppressive therapy with both CYC and MMF. Furthermore, a greater change in CXCL4 from baseline to 12 months led to improvements in SSc-ILD at 24 months as measured by the FVC %-predicted. These findings suggest that intermediate-term changes in CXCL4 may have predictive significance for long-term progression of SSc-ILD in patients receiving immunosuppressive therapy.

These findings are consistent with the purported pathobiological role of CXCL4 in moderating fibrotic and inflammatory processes. In addition to the previously reported association with various dimensions of SSc [16], CXCL4 levels have also been elevated in liver fibrosis [21] and in patients with antiphospholipid syndrome [22]. In a recent study, CXCL4 blocked the induction of the anti-inflammatory enzyme heme oxygenase-1. Furthermore, the product of heme oxygenase enzymatic activity, bilirubin, showed an inverse correlation with CXCL4 levels in plasma samples of SSc patients [23]. While our understanding of the role of CXCL4 in the development of SSc-ILD is evolving, the present manuscript provides further support for its role in the pathogenesis of SSc.

This study further sheds light on plausible biological mediators/targets of the two immunosuppressant agents under study (i.e. MMF and CYC). Although CXCL4 levels were not correlated with any of the baseline surrogate markers of ILD severity, CXCL4 levels significantly declined in both treatment arms in response to immunosuppression. Furthermore, the decline in CXCL4 paralleled the improvements appreciated in the course of the FVC over 24 months as reported in the main SLS II manuscript [19]. Although there was no significant difference in the degree to which CXCL4 decreased between treatment arms, the sample size may have limited our ability to detect a difference because CXCL4 levels appeared to decrease to a greater extent in the CYC arm compared with the MMF arm within the first 12 months of treatment (Fig. 3). Interestingly, CXCL4 levels declined most dramatically during the first 12 months of therapy in both study arms. The patients assigned to CYC received placebo in the second year, which may explain the slight upward trend in CXCL4 levels during the second year of the study. However, the CXCL4 levels did not return to those observed at the baseline visit after 2 years in the CYC arm. This finding may in part be related to the use of alternate therapy during the latter period. Of the 15 subjects in SLS II who withdrew from or failed treatment but returned for final study outcome measures, 12 indicated that they were switched to potentially disease-modifying therapy (MMF, intravenous CYC, rituximab, tocilizumab, intravenous immunoglobulin, or azathioprine) and these alternative therapies may have influenced the results [19]. In the MMF arm, CXCL4 levels continued to decline during the second year of the study, likely related to continuous use of immunosuppression in this arm.

Interestingly, we did not observe significant correlations between baseline CXCL4 levels and extent of ILD as measured by the FVC, DLCO, TLC, or extent of quantitative radiographic fibrosis at baseline, 12 months, or 24 months. While a prior study [16] demonstrated that patients with high CXCL4 were more likely to have fibrosis on HRCT, this prior observational study included SSc patients both with and without ILD and at varying disease stages. By contrast, the present study only included patients with ILD at a relatively early stage of disease (mean disease duration of 2.6 years from the onset of the first non-Raynaud’s symptom attributable to SSc), nearly all of whom already had evidence of fibrosis on HRCT [19]. Our findings suggest that CXCL4 may be a more useful predictive biomarker for response to immunosuppression in patients with early SSc-ILD. Whether CXCL4 correlates with severity of ILD at later stages of SSc remains uncertain. Furthermore, because our cohort did not evaluate SSc patients without ILD, we could not assess whether CXCL4 levels can distinguish SSc patients with and without ILD.

We did observe a significant but weak correlation between CXCL4 levels and platelet levels. However, the platelet level did not demonstrate predictive significance for FVC progression in the mixed-effects model. Importantly, precautions were taken to limit platelet activation, which can significantly affect CXCL4 levels, including using strict caution to minimize venostasis during the blood draw with butterfly needles and immediately centrifuging the specimens. Of note, the prior study in this area [16] did not assess platelet levels.

Consistent with the work of van Bon et al. [16], we observed an increase in plasma CXCL4 levels in SSc-ILD patients compared with healthy controls. However, the magnitude of this difference was strikingly smaller in the present study (1.6-fold increase in our study vs 277-fold increase in the previous study). The observed difference in CXCL4 levels between SSc-ILD patients and healthy controls in the present report is consistent with the reported fold increases of other cytokines and chemokines in SSc plasma samples [12, 24]. While several possible explanations for this disparity exist, we note that we used a different ELISA assay in the present study than in the previous study (R&D Systems/Quantikine in the present study vs R&D Systems/DuoSet in the previous study). The assay we utilized has been fully validated for the measurement of CXCL4 in complex fluids such as serum and plasma [25].

There are notable strengths of the present study. First, this is the first study to measure CXCL4 levels at multiple time points in patients with early, symptomatic SSc-ILD. Second, our initial measurement of CXCL4 occurred prior to the initiation of immunosuppressive therapy for SSc-ILD; thus, the reported baseline levels of CXCL4 are likely closely related to the underlying disease state without any interference from disease-modifying therapies (patients were ineligible for SLS II if they had received CYC or MMF for >8 weeks ever in the past, or they had received intravenous CYC more than twice ever in the past, or were taking CYC/MMF/any medication with potential disease-modifying activity within 30 days of randomization). Third, unlike prior observational studies in this field, this cohort had uniform follow-up measurements with likely less missing data. Fourth, our analyses controlled for treatment assignment. Finally, this study was conducted at 14 centers and included patients from varying ethnic backgrounds, thereby increasing the generalizability of our findings.

The results of the present study should be interpreted within the context of certain limitations. Namely, although we attempted to control for treatment group, some patients in the SLS II cohort initiated alternate therapy for SSc-ILD during the course of the trial. Given the relatively small number of patients who switched therapies and the observation that there was no difference in the change in CXCL4 between the two treatment groups, it is unlikely that the use of alternate therapies would alter the outcomes of the present analyses. Another shortcoming was that only patients who returned for a 12-month and 24-month visit provided blood samples. The missing CXCL4 data reduce the statistical power of the analysis and may yield biased estimates.

Future studies may consider measuring CXCL4 at even earlier time points as a means of determining treatment response prior to 1 year. While we report that the change in CXCL4 at 1 year was associated with improved SSc-ILD progression at 2 years, additional studies would be useful to determine whether changes in CXCL4 at earlier time points might have similar predictive value. If the latter were found, it might be useful from a clinical standpoint to determine whether a patient is responding to a particular therapy at 3 or 6 months, to avoid the unfruitful continuation of an ineffective therapy.

## Conclusions

While CXCL4 was not associated with extent of SSc-ILD at baseline, this chemokine decreased significantly in response to immunosuppressive therapy. Moreover, the change in CXCL4 levels was associated with future progression of SSc-ILD. Although the exact role of CXCL4 in the pathobiology of SSc-ILD is uncertain, these findings support the potential predictive capability of this chemokine for determining response to immunosuppressive therapy.

## Abbreviations

CAD: Computer-aided design
CCL18: CC chemokine ligand 18
CRP: C-reactive protein
CYC: Cyclophosphamide
DL/VA: Ratio of DLCO to alveolar volume
DLCO: Diffusing capacity for carbon monoxide
FEV_1_: Forced expired volume in 1 second
FRC: Functional residual capacity
FVC: Forced vital capacity
GGO: Ground glass opacity
HAQ: Health Assessment Questionnaire
HRCT: High-resolution chest computed tomography
IL: Interleukin
ILD: Interstitial lung disease
LM: Lobe of maximal involvement
MCP-1: Monocyte chemoattractant protein-1
MMF: Mycophenolate
mRSS: Modified Rodnan skin score
QILD: Quantitative extent of total interstitial lung disease (including fibrosis, honeycomb, and ground glass opacity)
QLF: Quantitative extent of lung fibrosis on HRCT
RV: Residual volume
SLS: Scleroderma Lung Study
SSc: Systemic sclerosis
TLC: Total lung capacity
WL: Whole lung
